# THItoGene: a deep learning method for predicting spatial transcriptomics from histological images

**DOI:** 10.1093/bib/bbad464

**Published:** 2023-12-25

**Authors:** Yuran Jia, Junliang Liu, Li Chen, Tianyi Zhao, Yadong Wang

**Affiliations:** Institute for Bioinformatics, School of Computer Science and Technology, Harbin Institute of Technology, Harbin, 150040, China; Institute for Bioinformatics, School of Computer Science and Technology, Harbin Institute of Technology, Harbin, 150040, China; School of Life Sciences, Westlake University, Hangzhou, Zhejiang 310024, China; School of Medicine and Health, Harbin Institute of Technology, Harbin, 150040, China; School of Medicine and Health, Harbin Institute of Technology, Harbin, 150040, China

**Keywords:** spatial transcriptomics, histopathological images, transformer, capsule network

## Abstract

Spatial transcriptomics unveils the complex dynamics of cell regulation and transcriptomes, but it is typically cost-prohibitive. Predicting spatial gene expression from histological images via artificial intelligence offers a more affordable option, yet existing methods fall short in extracting deep-level information from pathological images. In this paper, we present THItoGene, a hybrid neural network that utilizes dynamic convolutional and capsule networks to adaptively sense potential molecular signals in histological images for exploring the relationship between high-resolution pathology image phenotypes and regulation of gene expression. A comprehensive benchmark evaluation using datasets from human breast cancer and cutaneous squamous cell carcinoma has demonstrated the superior performance of THItoGene in spatial gene expression prediction. Moreover, THItoGene has demonstrated its capacity to decipher both the spatial context and enrichment signals within specific tissue regions. THItoGene can be freely accessed at https://github.com/yrjia1015/THItoGene.

## INTRODUCTION

With the development of many spatial transcriptomics techniques that can align high-resolution hematoxylin and eosin (H&E)-stained histological images with high-throughput ribonucleic acid (RNA) sequencing, we have been able to systematically characterize cell types and states while preserving positional information [1–6]. Spatial transcriptomics technology has revolutionized our understanding of tissue structure and function by correlating cellular functionality, morphology and location with gene expression. It offers a novel perspective that facilitates the discovery of information pertaining to cell–cell interactions and molecular signaling. In the field of oncology, spatial transcriptomics enables unbiased analysis of cellular states, positions and potential interactions within the tumor microenvironment, allowing for the exploration of transcriptional differences among different pathological features of tumors [7–9]. This is of great significance for comprehensively understanding the molecular mechanisms underlying tumor development, achieving more accurate molecular subtyping of patients and discovering spatially related biomarkers. Unfortunately, the high cost of data generation limits the application of spatial transcriptome technology in large-scale studies [10].

High-resolution histological images contain rich yet underutilized biomedical signals. Previous studies have shown that abnormal gene expression or mutations often affect cell morphology, structure, and distribution, leading to changes in histological features [11, 12]. By utilizing methods such as computer vision, we can establish associations between pathological images and the occurrence and development of diseases, thereby enabling quantitative analysis of biological features such as cellular structures, protein expression and gene expression [13–15]. Jain *et al.* [16] proposed Image2TMB, a multi-scale deep learning model for detecting global morphological changes caused by aggregated mutations in individual tumor cells in routine histopathology images. MOMA is a scalable deep learning method for predicting alterations in clinical molecular events in cancer genomics, proteomics and patient prognosis developed by Yu *et al*. [17]. Wagner *et al.* [18] combined a pre-trained Transformer encoder with a Transformer network for patch aggregation to predict biomarkers from colorectal cancer histology.

Furthermore, existing methodologies have demonstrated the ability to learn specific molecular characterization from histological patterns. HE2RNA [19] employs deep learning to reconstruct transcriptomic maps from histological images, showcasing its utility in clinical diagnosis. Pathological images furnish valuable information regarding tissue structure, cellular morphology and pathological features, while spatial transcriptomics techniques reveal the spatial distribution of genes and their expression patterns within tissues. Integrating these two approaches can offer a more comprehensive understanding and provide deeper insights for pathological research and disease diagnosis. Compared to the expensive ST technology, extracting molecular features from tissue section images stained with H&E is a faster and more cost-effective alternative [10, 20]. ST-Net [21] successfully combines cellular morphology with gene expression, enabling prediction from histology to spatial transcriptomics. Li *et al.* [22] developed a method named HisToGene based on the Vision Transformer (ViT), which can predict super-resolution gene expression in tumor histology images. However, ST-Net only extracts image features through convolutional neural networks (CNNs) and does not incorporate spatial positional information of gene expression during modeling, it fails to learn the associations between gene expression in spatially adjacent positions. HisToGene improves upon the existing ViT by considering both the positional information of gene expression and histological features. However, since ViT requires compressing the image into sequences for input despite the addition of spatial positional encoding in the enhanced ViT, there is still a certain degree of information loss. His2ST [10] addresses the issue of spatial relationship loss in HisToGene by explicitly learning the neighborhood relationships of histological features using a combination of CNNs and graph convolutional networks. Nevertheless, histopathology images contain a large number of morphologically diverse cellular landscapes and complex pathological features. Current methods lack the necessary flexibility and depth to interpret the complex structures found in histopathology images and are unable to effectively resolve detailed features at super-resolution, resulting in insufficient accuracy in predicting spatial gene expression.

To overcome the limitations of existing methods, we propose a novel deep learning framework called THItoGene. THItoGene is designed to accurately predict gene expression at a spatial resolution in pathology images by integrating spatial information and histological multi-view neighborhood features. Comprehensive benchmarking on spatial transcriptomic data generated from different tumors demonstrates the exceptional predictive capability of THItoGene. Moreover, THItoGene has exhibited its capability in recognizing significant alterations in gene expression within pathologist-annotated spatial domains and predicting the enrichment of specific genes in complex biological tissues.

## MATERIALS AND METHODS

### Overview of THItoGene

**Figure 1:**
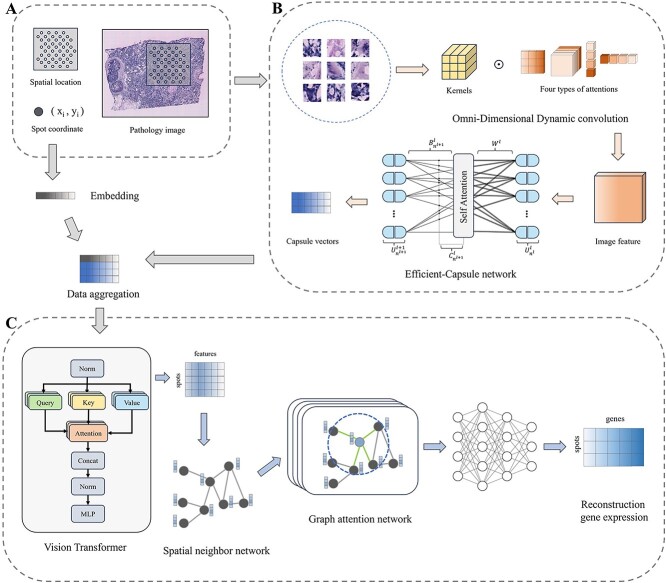
Overview of THItoGene. (**A**) Segmentation of the histological image into patches corresponding to each spot’s location and creation of position embeddings for the spatial coordinates of each spot. (**B**) Extraction of deep molecular features from patches utilizing the dynamic convolution module and the Efficient-Capsule module. (**C**) Global modeling of position embeddings and pathology features using the ViT module to understand cross-positional association relationships in the images, followed by adaptive learning of the relationship between the spatial position of the neighborhood of the spot and gene expression based on the GAT module.

THItoGene is specifically designed to provide a comprehensive representation of the intricate relationships between gene expression, spatial location and histopathological images (Figure 1). To characterize super-resolution histopathology images, THItoGene first leverages the multidimensional attention mechanism of dynamic convolution to extract comprehensive and detailed local visual information from the initial input image. Compared to conventional convolutions, dynamic convolutional networks are able to effectively capture complex, detailed features and enhance the perception of specific cellular landscape textures through more flexible tuning capabilities. The Efficient-CapsNet module captures the spatial relationships and distribution patterns of cells through the dynamic routing mechanism, learning the spatial arrangement and interaction information of different types of cells, thereby enhancing the understanding and prediction of gene expression.

THItoGene then fuses the spot location information embedding and the depth image features learned from the histological images to perform global modeling via the ViT module to comprehend the correlation between different regions. Finally, THItoGene constructs a neighborhood network based on the relative spatial positions between spots and adaptively characterizes the relationship between the spatial position of spots and gene expression using the graph attention network (GAT) module.

### Datasets

In the evaluation process of THItoGene, we utilized spatial transcriptomics data from two different tumors generated by the 10X Genomics platform: human HER2 positive breast cancer (HER2+) [23] and human cutaneous squamous cell carcinoma (cSCC) [24] datasets. In the HER2+ dataset, a total of 36 sections from eight breast cancer patients were included. We retained 32 sections with a spot count greater than 180 to assess the performance of THItoGene. In the cSCC dataset, there were 12 tissue sections from four patients included.

For the spatial transcriptomic gene expression data of the two tumors, we filtered out genes that were expressed in fewer than 1000 spots across all tissue sections and only considered the top 1000 highly variable genes expressed in the sections [22]. We then normalized the gene feature counts within each spot by dividing them by the total feature counts of all genes, multiplied by 1 000 000, and applied a natural logarithmic transformation. After processing, the HER2+ dataset contained 9612 spots and 785 genes, while the cSCC dataset contained 6630 spots and 134 genes. When processing the pathological images, we divided the entire image into N patches of size (3*W*H), where W and H represent the width and height of each patch. To match the diameter of the spots, we set W and H to 112 pixels.

### Dynamic convolution module

Compared to conventional convolution, which employs a fixed convolution kernel to process various locations of a histological image uniformly, dynamic convolution has the capability to adaptively adjust the size of the convolution kernel according to different spatial locations. This allows for the enhanced capturing and representation of the multi-scale fine features found in diverse cellular and tissue structures within the histological image. The process can be illustrated as follows:


(1) \begin{equation*} Y=\left({\alpha}_{w1}{W}_1+{\alpha}_{w2}{W}_2+\dots +{\alpha}_{wn}{W}_n\right)\ast X \end{equation*}

where $X\in{R}^{w\times h\times{c}_{in}}$ and $Y\in{R}^{w\times h\times{c}_{out}}$ represent the input image and the extracted features, respectively; ${W}_i\in{R}^{k\times k\times{c}_{in}}$ represents the $i$th convolutional kernel; ${\alpha}_{wi}\in R$ denotes the dynamic attention coefficients; $\ast$ stands for convolutional operations.

Omni-dimensional Dynamic Convolution [25] is a design of dynamic convolution that learns different attention weights along the four dimensions of convolutional kernel space, including kernel size ($k\times k$), input channel number(${c}_{in}$), output channel number(${c}_{\mathrm{out}}$), and the number of convolutional kernels($n$), in order to better adapt to variations in input features:


(2) \begin{align*} Y=\left({\alpha}_{w1}\odot{\alpha}_{k1}\odot{\alpha}_{c1}\odot{\alpha}_{o1}\odot{W}_1+\dots\right.\nonumber\\ \ \ \left. +\ {\alpha}_{wn}\odot{\alpha}_{kn}\odot{\alpha}_{cn}\odot{\alpha}_{on}\odot{W}_n\right)\ast X \end{align*}

In this equation, ${\alpha}_{wi}\in R$ represents the attention coefficient of the convolutional kernel ${W}_i$. ${\alpha}_{ki}\in{R}^{k\times k}$, ${\alpha}_{ci}\in{R}^{c_{in}}$ and ${\alpha}_{oi}\in{R}^{c_{out}}$, respectively, denote the attention weights computed along the spatial dimensions of the convolutional kernel ${W}_i$, the input channel dimension, and the output channel dimension. The symbol $\odot$ represents element-wise multiplication across different dimensions of the kernel space. The ${\alpha}_{wi}$, ${\alpha}_{ki}$ , ${\alpha}_{ci}$ and ${\alpha}_{oi}$ are computed using the multi-head attention module ${\varphi}_i(x)$. In ${\varphi}_i(x)$, the input $x$ is first compressed into a feature vector of length ${c}_{\mathrm{in}}$ through the Global Average Pooling operation. Then, the compressed feature vector is mapped to a lower-dimensional space using a fully connected (FC) layer. Finally, for each attention head branch, there is an output FC layer of size $n\times 1,k\times k,\kern0.5em {c}_{\mathrm{in}}\times 1$ and ${c}_{\mathrm{out}}\times 1$, as well as a Sigmoid function, used to generate normalized attention coefficients ${\alpha}_{wi},\kern0.5em {\alpha}_{ki}$, ${\alpha}_{ci}$ and ${\alpha}_{oi}$.

### Efficient-CapsNet module

The Efficient-CapsNet [26] module is an optimized version of the Capsule Network that utilizes self-attention mechanisms to effectively route capsules, significantly reducing the number of model parameters. By employing dynamic routing mechanisms, the Efficient-CapsNet enables communication and collaboration among capsules. The dynamic routing allocates weights between capsules based on their similarities, thereby determining the final output feature representation. This mechanism helps capture spatial relationships and hierarchical structures among features, allowing for the learning of multi-level feature representations in pathological images and modeling the spatial relationships between features. In the Efficient-CapsNet, the input is first mapped to a high-dimensional space:


(3) \begin{equation*} {\displaystyle \begin{array}{c}{F}^{l+1}\left({X}^l\right)=\mathrm{Relu}\left(\mathrm{Con}{v}_{k\times k}\left({X}^l\right)\right)\end{array}} \end{equation*}

The shape of ${X}^l$ is represented as $W\times H\times C$*,* where $W$, $H$ and $C$ denote the width, height, and number of channels of the convolved image, respectively. The convolutional kernel has a size of $k\times k$ and a stride of $s=1$. Subsequently, $C$ convolutional kernels, each of the same size as in Eq. (4), are applied to each channel independently, resulting in the primary capsule layer denoted as ${S}_{n,d}^l$, where $n$ and $d$ represent the number of primary capsules in the $l$th layer and the dimensionality of a single capsule, respectively.

After this, it is activated by the squash operation and routed to the whole by the self-attention routing algorithm:


(4) \begin{equation*} {\displaystyle \begin{array}{c}{u}_n^l=\mathrm{squash}\left({s}_n^l\right)=\left(1-\frac{1}{e^{\left\Vert{s}_n^l\right\Vert }}\right)\frac{s_n^l}{\left\Vert{s}_n^l\right\Vert}\end{array}} \end{equation*}

(5) \begin{equation*} {\displaystyle \begin{array}{c}{U}_{n^{l+1}}^l={u}_n^l\times{W}^l\end{array}} \end{equation*}

(6) \begin{equation*} {\displaystyle \begin{array}{c}{s}_n^{l+1}={\left({U}_{n^{l+1}}^l\right)}^T\times \left({C}_{n^{l+1}}^l+{B}_{n^{l+1}}^l\right)\end{array}} \end{equation*}

where ${s}_n^l$ and ${u}_n^l$ represent the capsules before and after activation, respectively; ${B}_{n^{l+1}}^l$ represents the log priors matrix; ${U}_{n^{l+1}}^l$ represents the prediction of all primary capsules in the $l$th layer, and ${W}^l$ is used for the prediction’s weight matrix. ${C}_{n^{l+1}}^l$ represents the matrix of all coupling coefficients generated after self-attention, and it is summed with ${B}_{n^{l+1}}^l$ with to get the final routing weights. The coupling coefficient ${C}_{n^{l+1}}^l$ can be expressed as:


(7) \begin{equation*} {\displaystyle \begin{array}{c}{C}_{n^{l+1}}^l=\frac{\exp \left({\sum}_{n^l}\frac{U_{n^{l+1}}^l\times{\left({U}_{n^{l+1}}^l\right)}^T}{\sqrt{d^l}}\right)}{\sum_{n^{l+1}}\exp \left({\sum}_{n^l}\frac{U_{n^{l+1}}^l\times{\left({U}_{n^{l+1}}^l\right)}^T}{\sqrt{d^l}}\right)\ }\end{array}} \end{equation*}

where ${d}^l$ represents the dimension of each capsule in the $l$th layer, and ${U}_{n^{l+1}}^l$ comes from the calculation of Eq. (6).

### ViT module

Subsequently, we encode the spatial location information coordinates$\left(x,y\right)$ of spots into the feature matrices of ${E}_x\in{R}^{1\times d}$ and ${E}_y\in{R}^{1\times d}$, respectively, and fuse them with the visual features of the pathology images, ${S}^{l+1}\in{R}^{n\times d}$, and use ViT to capture the long-range dependencies in the images:


(8) \begin{equation*} {\displaystyle \begin{array}{c}{S}^{l+2}=\mathrm{concat}\left({S}^{l+1},{E}_x,{E}_y\right)\end{array}} \end{equation*}

where ${S}^{l+1}\in{R}^{n\times d}$ is the output of the capsule network, $n$ and $d$ are the number and dimension of the output capsule, respectively; and then the attention of each spot is learned through the multi-head attention layer of the Transformer, which assigns different weights to different image patches:


(9) \begin{equation*} {\displaystyle \begin{array}{c}\mathrm{Attention}\left(Q,K,V\right)=\mathrm{softmax}\left(\frac{Q{K}^T}{\sqrt{d_k}}\right)V\end{array}} \end{equation*}

(10) \begin{equation*} {\displaystyle \begin{array}{c}\mathrm{hea}{\mathrm{d}}_i=\mathrm{Attention}\left(X{W}_i^Q,X{W}_i^K,X{W}_i^v\right)\end{array}} \end{equation*}

(11) \begin{equation*} {\displaystyle \begin{array}{c}H(X)=\mathrm{concat}\left({\mathrm{head}}_1,{\mathrm{head}}_2,\dots, \mathrm{hea}{\mathrm{d}}_m\right)\times{W}^h\end{array}} \end{equation*}

where $Q,K,V$ represent ‘Query’, ‘Key’ and ‘Value’, respectively; ${W}_i^Q$、${W}_i^K$ and ${W}_i^v$ are the weight matrices for transforming inputs into $Q,K,V$; The term $\frac{Q{K}^T}{\sqrt{d_k}}$ represents the similarity between Query, Key and the Attention coefficient can be obtained by weighting and summing the values of each $V$ by the weight coefficients calculated by Softmax. ${W}^h$ is the weight matrix of multiple Attention heads, and $m$ is the number of Attention heads.

### Graph attention network module

To explore the correlation between gene expression and spatial location, THItoGene uses GAT to explicitly learn the interactions between spatially located neighboring spots. GAT could pass information in a specific network structure, learn different attention values based on the importance of each neighbor of the node, and then use these weights to aggregate the features of the neighbors.

We refer to His2ST [10] for the nearest neighbor graph construction. The spatial locations of the Euclidean distances are used as edges in the nearest neighbor graph by calculating the Euclidean distances. Specifically, we select four nearest neighbors for each spot to construct the nearest neighbor graph $G=\left(V,E\right)$. $N=\left|V\right|$ denotes the number of spots, and $E$ denotes the edges connected to the nearest neighbors. The Euclidean distance between every two spots $\left(i,j\right)$ is calculated as follows:


(12) \begin{equation*} {\displaystyle \begin{array}{c}d\left(i,j\right)=\sqrt{{\left({i}_x-{j}_x\right)}^2+{\left({i}_y-{j}_y\right)}^2}\end{array}} \end{equation*}

We then use GAT to aggregate the node features in the network. Here, the importance of the $j$th node with respect to the $i$th node is calculated as follows:


(13) \begin{equation*} {\displaystyle \begin{array}{c}{e}_{ij}=f\left({W}_h{h}_i,{W}_h{h}_j\right)\end{array}} \end{equation*}

where $f$ is a single-layer feed-forward neural network, ${W}_h$ is a learnable parameter matrix which transforms the input features into a hierarchical feature representation between spots, and ${h}_i\in{R}^M$ is the feature representation of the $i$th node. Here, THItoGene unfolds the features output from ViT into a one-dimensional vector of length $M$. In order to regulate the influence between different nodes, the importance weights are normalized among all neighboring nodes of ${v}_i$:


(14) \begin{equation*} {\displaystyle \begin{array}{c}{\alpha}_{ij}=\frac{\exp \left(\mathrm{LeakyRelu}\left({e}_{ij}\right)\right)}{\sum_{t\in \varOmega \left({v}_i\right)}\exp \left(\mathrm{LaekyReLU}\left({e}_{it}\right)\right)}\end{array}} \end{equation*}

where $\varOmega \left({v}_i\right)$ represents the neighboring nodes of ${v}_i$ and the normalized attention coefficients are used to compute a linear combination of the features of the neighboring nodes:


(15) \begin{equation*} {\displaystyle \begin{array}{c}{h}_{\varOmega \left({v}_i\right)}=\sum_{t\in \varOmega \left({v}_i\right)}{\alpha}_{it}{h}_t\end{array}} \end{equation*}

Finally, we connect the current node feature representation with the feature representations of its neighboring nodes to update the embedding:


(16) \begin{equation*} {\displaystyle \begin{array}{c}h=\mathrm{Elu}\left(\mathrm{concat}\left({h}_{\ast },{h}_{\varOmega \left(\ast \right)}\right)\ \omega +\beta \right)\end{array}} \end{equation*}

where ω and β are the parameterized weight and bias matrices, respectively.

### Evaluated metrics and criteria

In this study, we employ the Pearson correlation coefficient (PCC) to compare the spatial gene expression predicted by THItoGene with the observed gene expression, in order to assess their level of correlation. The PCC has a range of values between −1 and 1. It is calculated by dividing the covariance of the two variables by the product of their respective SDs:


(17) \begin{equation*} {\displaystyle \begin{array}{c}\mathrm{PCC}=\frac{\mathrm{Cov}\left({X}_{\mathrm{true}},{X}_{\mathrm{pred}}\right)}{\sigma \left({X}_{\mathrm{true}}\right)\cdot \sigma \left({X}_{\mathrm{pred}}\right)}\end{array}} \end{equation*}

where $\mathrm{Cov}$ denotes covariance; ${X}_{\mathrm{true}}$ and ${X}_{\mathrm{pred}}$ denote the original gene expression and the gene expression obtained by prediction, respectively. $\sigma \left(\right)$ denotes SD.

When evaluating the performance of spatial clustering, we employ the Adjusted Rand Index (ARI) to measure the similarity between the clustering results and the true pathological annotation regions. It has a range of values between −1 and 1, where a value closer to 1 indicates a higher consistency between the clustering results and the true labels. Conversely, a value closer to 0 suggests that the similarity between the clustering results and the true labels is random, while a value closer to −1 indicates a complete opposition between the clustering results and the true labels. The ARI can be expressed as follows:


(18) \begin{equation*} {\displaystyle \begin{array}{c}\mathrm{ARI}=\frac{\sum_{ij}\left(\begin{array}{c}{n}_{ij}\\{}2\end{array}\right)-\left[{\sum}_i\left(\begin{array}{c}{n}_i\\{}2\end{array}\right){\sum}_j\left(\begin{array}{c}{n}_j\\{}2\end{array}\right)\right]/\begin{array}{c}n\\{}2\end{array}}{\frac{1}{2}\left[{\sum}_i\left(\begin{array}{c}{n}_i\\{}2\end{array}\right)+{\sum}_j\left(\begin{array}{c}{n}_j\\{}2\end{array}\right)\right]-\left[{\sum}_i\left(\begin{array}{c}{n}_i\\{}2\end{array}\right){\sum}_j\left(\begin{array}{c}{n}_j\\{}2\end{array}\right)\right]/\begin{array}{c}n\\{}2\end{array}}\end{array}} \end{equation*}

where ${n}_i$ and ${n}_j$ denote the number of samples appearing in the $i$th prediction cluster and the $j$th true cluster, respectively; ${n}_{ij}$ denotes the number of overlapping samples between the $i$th prediction cluster and the $j$th true cluster.

## RESULT

### THItoGene can improve the prediction accuracy of spatial resolution gene expression

**Figure 2:**
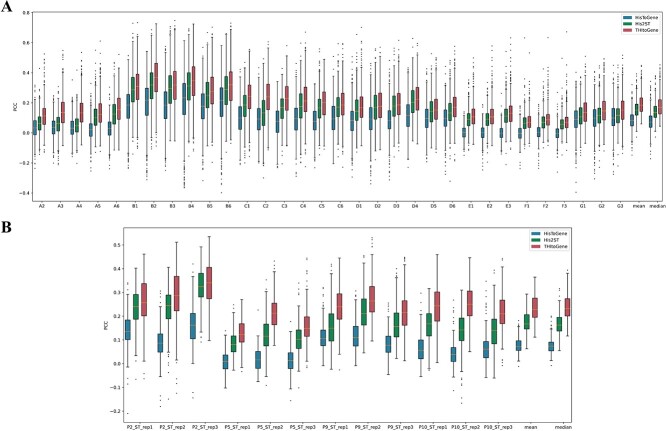
Comparative evaluation of spatial transcriptome prediction methods. (**A**) Boxplot representation of PCC distributions comparing observed and predicted gene expressions in the HER2+ dataset. (**B**) Similar PCC distribution comparisons for the cSCC dataset. The predictions are made by three different methods: THItoGene, His2ST and HisToGene.

In order to quantitatively evaluate the gene expression prediction performance of THItoGene, we first applied it to the HER2+ and cSCC datasets using leave-one-out cross-validation. We compared THItoGene to two other recently developed state-of-the-art methods for predicting spatial gene expression, HisToGene and His2ST. The comparison was based on calculating the PCC between predicted and observed gene expression in each tissue section. This allowed us to evaluate how accurately each method could predict actual spatial gene expression patterns. By comparing the PCCs, we aimed to determine which of the three methods provided the most accurate spatial gene expression predictions overall. Since HisToGene [22] does not give the models, we retrained HisToGene based on the parameters provided by the authors. For each section in the HER2+ and cSCC datasets, we utilized the remaining sections to train THItoGene and evaluated the correlation between predicted gene expression and actual gene expression on the current section. As shown in Figure 2A, among all the sections in the HER2+ dataset, THItoGene predicts the highest PCC between spatially resolved gene expression and actual gene expression. Notably, HisToGene has 11 sections (A2–A6, E1–F3) among the 32 sections in the HER2+ dataset with average correlations floating around the near-zero level. Among them, the average correlation of E3 and F1 sections was negative, which might be caused by its failure to capture the association relationship between deep histologic features and spot gene expression.

THItoGene exhibited the highest PCC across all tissue sections in the cSCC dataset (as shown in Figure 2B). Notably, it showed the greatest improvement over existing methods in predicting gene expression correlation for section P10_ST_rep2. These results provide strong evidence that THItoGene can more accurately forecast the relationship between spatial and actual gene expression. By incorporating adaptive super-resolution image signatures, THItoGene effectively reveals intricate cellular landscapes in histology images, enabling more reliable and precise gene expression prediction. In summary, THItoGene’s use of enhanced image features leads to considerable benefits in predicting spatial gene expression compared to current methods.

### Investigating the impact of each module in THItoGene on the predicted gene expression results

**Figure 3:**
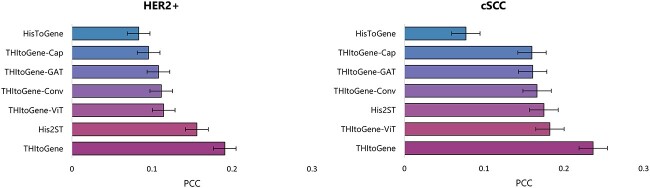
Ablation experiments on the HER2+ and cSCC datasets. The data showed the average PCC for THItoGene (after removing specific modules), HisToGene and His2ST in each dataset.

In order to comprehend the reasons behind the enhanced performance of THItoGene compared to other methods, we evaluated the contribution of each module to THItoGene. To achieve this, we removed key modules of THItoGene for the ablation experiment, as shown in Figure 3. Keeping all modules intact leads to the highest correlation between predicted and observed gene expression. Notably, excluding the Efficient-CapsNet module caused average PCC reductions of 0.095 and 0.076 for the HER2+ and cSCC datasets, only slightly higher than HisToGene’s performance. This suggests the Efficient-CapsNet module plays a key role in enabling THItoGene to uncover the relationship between histopathology and gene expression. Furthermore, removing the ViT, dynamic convolution or GAT modules also decreased performance to varying degrees. In summary, the results of the ablation experiment demonstrate the necessity of preserving the integrity of all modules in THItoGene to ensure optimal performance in predicting gene expression.

### THItoGene accurately predicts tumor-related genes

**Figure 4:**
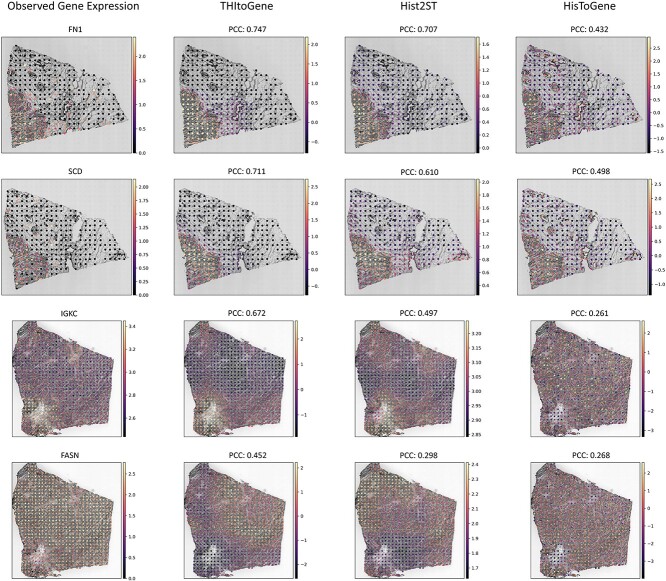
The top four predicted genes with the highest mean −log10 *P*-value in the HER2+ dataset. The first column displays the observed gene expression, while the last three columns show gene expression predictions from three different methods. We derived *P*-values for each gene by calculating the PCC between predicted and observed gene expressions. For visualization, we selected the section that exhibited the highest −log10 *P*-value for each gene.

We investigated whether THItoGene’s predicted gene expression reflects the true status of tumor-associated genes. In the HER2+ dataset, we analyzed the correlation between observed and predicted gene expression, calculating both correlation coefficients and *P*-values for each spot. We then derived the average −log10 *P*-value across all genes. These genes were ranked in descending order of their −log10 *P*-values, as detailed in Supplementary Table 1. We focused on the top four genes with the highest −log10 *P*-values (FN1, SCD, IGKC and FASN), which are visualized in Figure 4. THItoGene yielded correlation coefficients for these genes of 0.747, 0.711, 0.672 and 0.452, respectively—outperforming existing methods. Notably, these top-ranked genes have established associations with breast cancer, with studies indicating that the expression patterns of FN1 and IGKC correlate with patient survival and clinical outcomes [27, 28].

To demonstrate the scalability of THItoGene, we conducted same experiments on the cSCC dataset (Supplementary Table 2 and Supplementary Figure 1). THItoGene enables accurate prediction of key genes associated with cutaneous squamous cell carcinoma. This is in line with findings by Nindl *et al.* [29] and Zhu *et al*. [30] have both identified increased expression levels of NDRG1 and PI3 in cutaneous squamous cell carcinoma compared to normal skin.

### THItoGene effectively analyzes the changes in gene expression within the spatial region

**Figure 5:**
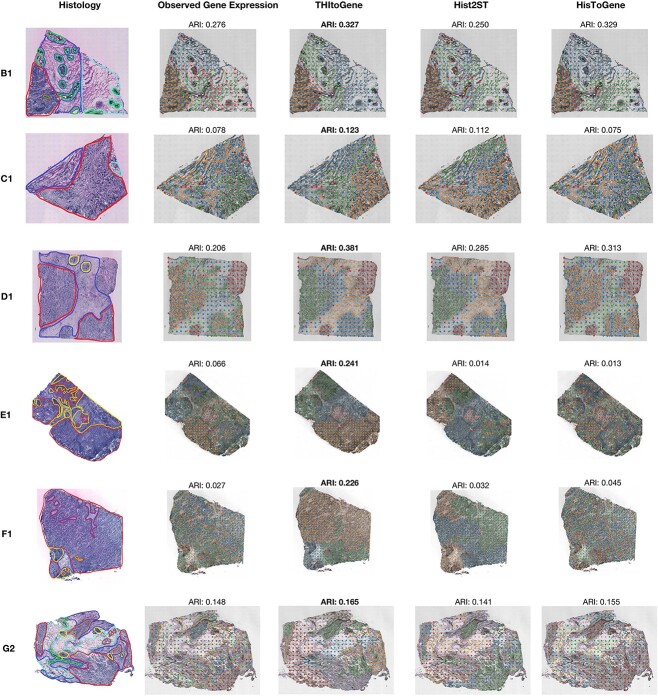
The spatial clustering analysis compares gene expression predictions from three different methods: THItoGene, Hist2ST and HisTGene. Histological images annotated by the pathologist are displayed in the first column. The second column presents the observed gene expression clustering results, while the last three columns show the clustering outcomes for gene expression as predicted by different methods.

The spatial gene expression predicted by THItoGene is capable of accurately restoring specific spatial domains. We performed quantitative analysis to evaluate the spatial clustering performance of gene expression predicted by THItoGene, using a set of six pathologist-annotated tissue sections (B1, C1, D1, E1, F1 and G2) from the HER2+ dataset. We used the spatial transcriptomics annotations generated by pathologists as the gold standard to assess the gene expression predicted by THItoGene, HisToGene and His2ST using K-means clustering (Figure 5). Compared to other methods, THItoGene is able to effectively identify the predefined spatial structures and achieve significant improvements. Specifically, in the B1 section, THItoGene (ARI = 0.327) achieved a similar ARI to HisToGene (ARI = 0.329) and significantly outperformed His2ST (ARI = 0.250). Across other sections, THItoGene consistently reconstructs spatial-specific gene expression more clearly. Interestingly, in all six sections, the clustering results obtained from the predicted gene expression by THItoGene outperformed those obtained from the observed gene expression. This discrepancy may stem from the inherent variability and noise in observed gene expression, affected by technical factors such as sequencing depth, batch effects and background noise, which can compromise clustering accuracy.

Compared to existing methods, THItoGene has the ability to capture the correlation between subtle differences in histopathological phenotypes and gene expression through dynamic convolutional adaptive learning. This allows for a better understanding of the spatial gene regulation patterns reflected by different histopathological features. Therefore, the gene expression data generated by THItoGene is superior in spatial domain identification and better reflects the true biological characteristics of tissues.

### THItoGene can predict differentially enriched molecular signals in complex tissue structures

**Figure 6:**
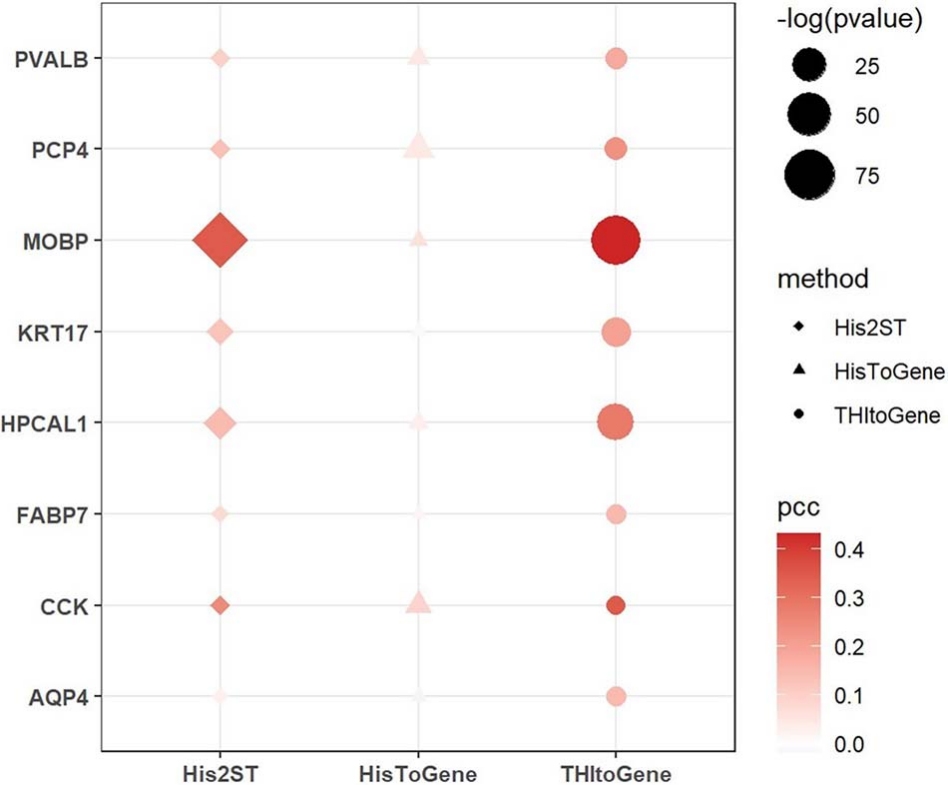
The results for layer marker genes predicted using three different methods within the spatial transcriptomics dataset of DLPFC.

We tested the ability of THItoGene to accurately predict context-specific signals within the complex structures of tissues. Specifically, we applied THItoGene to a spatial transcriptomics (ST) dataset of the human dorsolateral prefrontal cortex (DLPFC) obtained using 10x Visium technology [31]. This dataset included 12 sections from three different DLPFC samples. The DLPFC is functionally stratified into six layers, each with distinct neuronal compositions and roles in cognition, emotion regulation and decision-making [32]. The layered gene expression within the cortex is pivotal for the brain’s complex architecture. In our analysis, we assessed the accuracy of THItoGene in predicting the expression of genes enriched in specific layers by comparing it with His2ST and HisToGene, as shown in Figure 6 and detailed in Supplementary Table 3. THItoGene’s predictions more closely mirrored the actual gene expression patterns associated with different cortical layers. For instance, HPCAL1, recently identified by Kristen *et al.* [31] as a layer-specific marker gene in DLPFC’s layer 2, was precisely predicted by THItoGene in the corresponding brain regions.

## DISCUSSION

Spatial transcriptomics is an emerging technique that holds great promise for elucidating tissue development, understanding cell fate, advancing disease research, and facilitating drug discovery [33, 34]. Despite its promise, the high cost of spatial transcriptomics techniques limits their widespread application [35]. Computational predictions of spatial gene expression from histological images have emerged as a cost-effective alternative, yet existing methods often fail to capture the full complexity of molecular signatures. In this paper, we introduce THItoGene, a deep learning framework tailored for decoding spatial gene expression from pathology images. THItoGene utilizes histological images as input and employs dynamic convolutional and capsule networks to capture signals of potential molecular features within histological samples. In addition, it integrates spatial location information by utilizing ViT and GAT to establish a deep connection between spatial location and gene expression, thereby deciphering cellular landscapes in histopathology images.

However, THItoGene has not yet achieved the capability to resolve gene expression at the cellular or subcellular level with higher resolution. In future studies, our aim is to augment the feature characterization potential of THItoGene by employing active machine learning approaches. Furthermore, with the emergence of new technologies and the availability of more data, we anticipate that future research will enable us to achieve higher resolution prediction of spatial gene expression and enhance the overall performance of THItoGene.

THItoGene has demonstrated the ability to extract valuable molecular information from high-resolution histologic images containing hundreds of thousands of cells. By analyzing H&E-stained pathology images, the framework captures tissue structure, cell morphology, and lesion details, correlating them with gene expression. This is particularly valuable for research scenarios with scarce resources or tissue samples. THItoGene can facilitate the analysis of tumorigenesis and progression, enhance the understanding of complex interactions and spatial effects in the tumor microenvironment, and offer a robust tool for precision medicine in resource-limited settings.

**Key Points**

- We introduce THItoGene, a deep learning framework designed to predict spatially resolved gene expression profiles from histopathological images.
- THItoGene outperforms other methods in accurately predicting spatial gene expression and has demonstrated effectiveness across different tumor tissue sections.
- THItoGene is able to decipher spatial domains and enrichment signals within tissue regions.

## Supplementary Material

Supplementary_file_bbad464

supplementary_table_1_bbad464

supplementary_table_2_bbad464

supplementary_table_3_bbad464

## Data Availability

The human HER2-positive breast cancer datasets are available for download at https://github.com/almaan/her2st/. The human cutaneous squamous cell carcinoma dataset can be accessed through the GEO database under accession number GSE144240. The human dorsolateral prefrontal cortex dataset can be found at http://research.libd.org/spatialLIBD/.

## CODE AVAILABILITY

The source code for THItoGene is available in: https://github.com/yrjia1015/THItoGene. Trained models are available for download in: https://www.synapse.org/#!Synapse:syn52503858/files/

## Author Biographies

**Yuran Jia** is a PhD candidate at the School of Computer Science and Technology at the Harbin Institute of Technology of China. His research interests include bioinformatics.

**Junliang Liu** is a master candidate at the School of Computer Science and Technology at the Harbin Institute of Technology of China. His research interests include bioinformatics.

**Li Chen** is a postdoctoral researcher at the School of Life Sciences, Westlake University, with a focus on bioinformatics.

**Tianyi Zhao** is an associate professor at the School of Medicine and Health at the Harbin Institute of Technology of China. His research interests include molecular characterization of complex diseases, biological network analysis and machine learning.

**Yadong Wang** is a professor at the College of School of Medicine and Health at the Harbin Institute of Technology of China. His research interests are bioinformatics, machine learning and algorithms.
