# Supplementary material for: THItoGene: a deep learning method for predicting spatial transcriptomics from histological images
Source: Brief Bioinform. 2023 Dec 25;25(1):bbad464. doi: 10.1093/bib/bbad464 (PMC10749789; doi:10.1093/bib/bbad464)
Supplement: Supplementary_file_bbad464 [file supplementary_file_bbad464.docx]

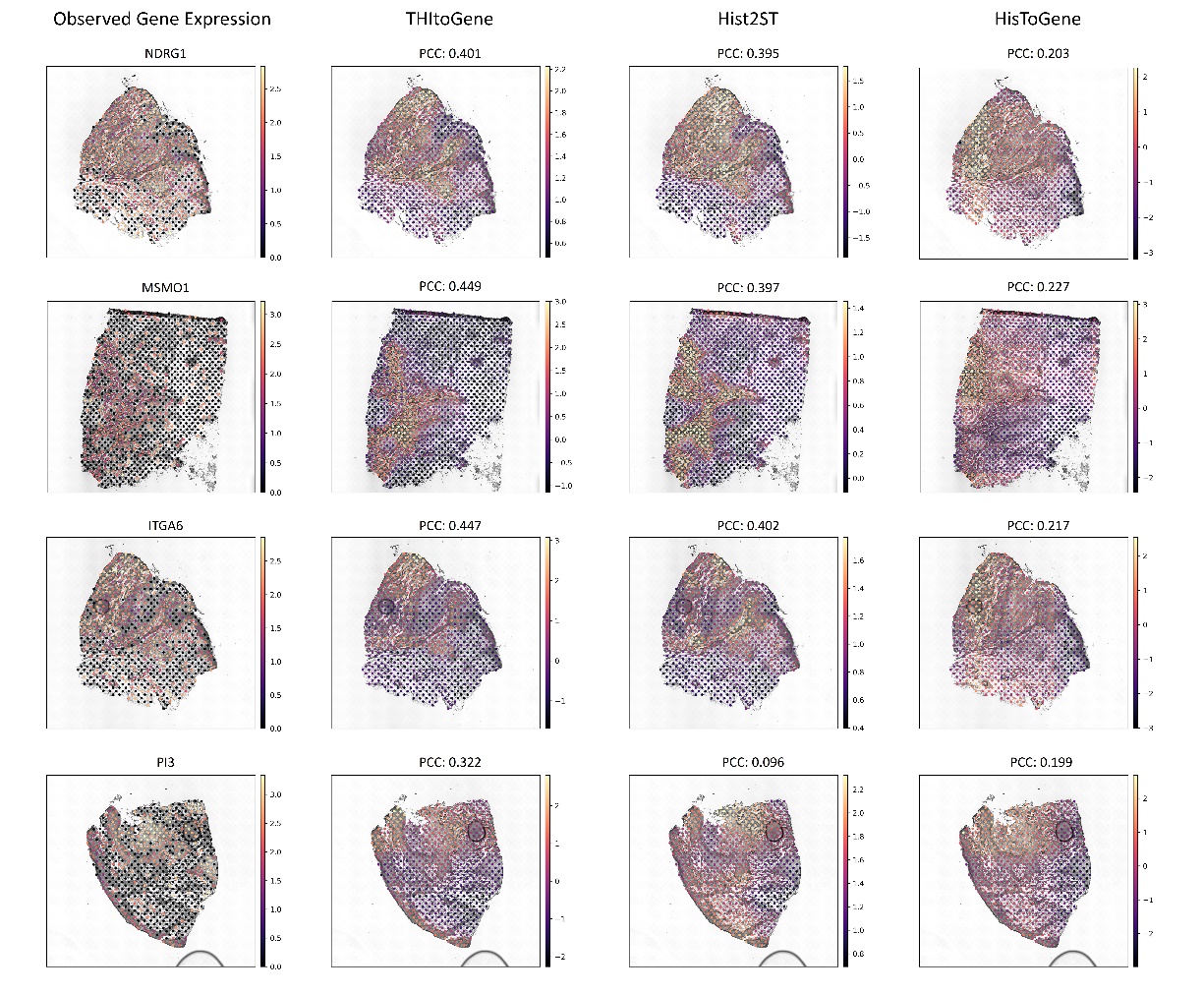


Supplementary Fig. 1 The top four predicted genes with the highest mean -log10 p-value in the cSCC dataset. The first column displays the observed gene expression, while the last three columns show gene expression predictions from three different methods. We derived p-values for each gene by calculating the PCC between predicted and observed gene expressions. For visualization, we selected the section that exhibited the highest -log10 p-value for each gene.
